# Genito-Urinary Diseases and Syphilis

**Published:** 1907-01

**Authors:** 


Genito-Urinary Diseases and Syphilis. By Henry H. Morton, M. D., Clinical Professor of Genito-Urinary Diseases in the Long Island College Hospital; Genito-Urinary Surgeon to the Long Island and Kings County Hospitals, and the Polhemus Memorial Clinic. Illustrated with 158 half-tone and photo-engravings and 7 full-page colored plates. Second edition, revised and enlarged. Royal octavo, 500 pages. Bound in extra cloth. Price, $4, net. F. A. Davis Co., Publishers, 1914-16 Cherry St., Philadelphia, Pa.

This I regard as a most timely work and a valuable addition to the literature of the subject. The illustrations alone are worth the price of the book. D.

